# The oral selective estrogen receptor degrader GDC-0810 (ARN-810) in postmenopausal women with hormone receptor-positive HER2-negative (HR + /HER2 −) advanced/metastatic breast cancer

**DOI:** 10.1007/s10549-022-06797-9

**Published:** 2022-11-19

**Authors:** Aditya Bardia, Ingrid Mayer, Eric Winer, Hannah M. Linden, Cynthia X. Ma, Barbara A. Parker, Meritxell Bellet, Carlos L. Arteaga, Sravanthi Cheeti, Mary Gates, Ching-Wei Chang, Jill Fredrickson, Jill M. Spoerke, Heather M. Moore, Jennifer Giltnane, Lori S. Friedman, Edna Chow Maneval, Iris Chan, Komal Jhaveri

**Affiliations:** 1 Massachusetts General Hospital Cancer Center, Harvard Medical School, Bartlett Hall Extension 237, 55 Fruit St, Boston, MA 02114 USA; 2 Vanderbilt-Ingram Cancer Center, Nashville, TN USA; 3 AstraZeneca, Gaithersburg, MD USA; 4 Dana-Farber Cancer Institute, Boston, MA USA; 5 Yale Cancer Center, New Haven, CT USA; 6 University of Washington, Seattle, WA USA; 7 Washington University School of Medicine, St. Louis, MO USA; 8 University of California San Diego Moores Cancer Center, San Diego, CA USA; 9 Vall d’Hebron Institute of Oncology, Barcelona, Spain; 10 UT Southwestern Simmons Comprehensive Cancer Center, Dallas, TX USA; 11 Genentech, Inc, South San Francisco, CA USA; 12 ORIC Pharmaceuticals, South San Francisco, CA USA; 13 Memorial Sloan Kettering Cancer Center, New York, Weill Cornell Medical College, New York, NY USA

**Keywords:** Selective estrogen receptor degrader, GDC-0810; HR +, HER2 −, Phase 1, Postmenopausal, Metastatic breast cancer

## Abstract

**Purpose:**

GDC-0810 (ARN-810) is a novel, non-steroidal, orally bioavailable, selective estrogen receptor degrader (SERD) that potentially inhibits ligand-dependent and ligand-independent estrogen receptor (ER)-mediated signaling.

**Methods:**

A phase Ia/Ib/IIa dose escalation, combination treatment with palbociclib or a luteinizing hormone-releasing hormone, and expansion study determined the safety, pharmacokinetics, and recommended phase 2 dose (RP2D) of GDC-0810 in postmenopausal women with ER + (HER2 −) locally advanced or metastatic breast cancer (MBC). Baseline plasma ctDNA samples were analyzed to determine the *ESR1* mutation status.

**Results:**

Patients (*N* = 152) received GDC-0810 100–800 mg once daily (QD) or 300–400 mg twice daily, in dose escalation, expansion, as single agent or combination treatment. Common adverse events regardless of attribution to study drug were diarrhea, nausea, fatigue, vomiting, and constipation. There was one dose-limiting toxicity during dose escalation. The maximum tolerated dose was not reached. GDC-0810 600 mg QD taken with food was the RP2D. Pharmacokinetics were predictable. FES reduction (> 90%) highlighting pharmacodynamic engagement of ER was observed. Outcomes for the overall population and for patients with tumors harboring *ESR1* mutations included partial responses (4% overall; 4% *ESR1*), stable disease (39% overall; 42% *ESR1*), non-complete response/non-progressive disease (13% overall; 12% *ESR1*), progressive disease (40% overall; 38% *ESR1*), and missing/unevaluable (5% overall; 5% *ESR1*). Clinical benefit (responses or SD, lasting ≥ 24 weeks) was observed in patients in dose escalation (*n* = 16, 39%) and expansion (*n* = 24, 22%).

**Conclusion:**

GDC-0810 was safe and tolerable with preliminary anti-tumor activity in heavily pretreated patients with ER + advanced/MBC, with/without *ESR1* mutations, highlighting the potential for oral SERDs.

*Clinical Trial and registration date* April 4, 2013. NCT01823835 .

**Supplementary Information:**

The online version contains supplementary material available at 10.1007/s10549-022-06797-9.

## Introduction

Breast cancer is the leading cause of cancer death in women worldwide, with more than 1,300,000 new cases and nearly 500,000 deaths annually [1]. Hormone receptor-positive (HR +) breast cancer, with tumor expression of the estrogen receptor (ER) and/or progesterone receptor (PR), is the most common form of the disease. Endocrine therapies that either suppress ER signaling or inhibit aromatase in the biosynthesis of estrogen serve as a major treatment strategy. Aromatase inhibitors with or without cyclin-dependent kinase (CDK 4/6) inhibitors are the recommended first-line treatment option for HR + metastatic breast cancers (MBC), although most tumors ultimately develop resistance to the therapeutic regimen. There is mounting evidence that acquisition of mutations in the *ESR1* gene is a major contributor toward aromatase inhibitor resistance [2–4]. *ESR1* mutations in the ligand-binding domain of the ER confer ligand independence to estrogen while retaining dependence on the ER pathway. Consequently, there is interest in selective estrogen receptor degraders (SERDs) that target the ER directly, regardless of the *ESR1* mutation status, to potentially inhibit ligand-dependent as well as ligand-independent ER-mediated signaling [5].

GDC-0810 (ARN-810) is a novel, non-steroidal, orally bioavailable SERD that binds to the ER to limit the activity of estrogen and also induces conformational changes leading to receptor degradation, thereby combating ligand-dependent as well as ligand-independent ER signaling in ER + breast cancer. In in vivo MCF-7 breast cancer cell studies, GDC-0810 fully antagonized the response of ER to estrogens and induced proteasomal degradation of ERα [6]. GDC-0810 has also induced tumor regression in tamoxifen-sensitive as well as in tamoxifen-resistant ER + breast cancer xenograft models [6, 7]. Based on preclinical data, we conducted a proof-of-concept phase Ia/Ib/IIa clinical study of GDC-0810 in women who were postmenopausal and with locally advanced or metastatic ER + (HER2 −) breast cancer.

## Patients and methods

### Study design

This was a multi-institutional phase Ia/Ib/IIa, open-label, dose finding, safety, pharmacokinetics, and proof-of-concept study of GDC-0810 in women with ER + metastatic breast cancer. Phase Ia employed a standard 3 + 3 dose-escalation scheme. Phase Ib was dose escalation as part of combination treatment. Phase IIa explored the recommended phase 2 dose (RP2D) from phase Ia. The protocol was approved by institutional review boards (IRBs) at participating institutions. Written informed consent was obtained from patients prior to performing any procedures; the study was conducted in accordance with the principles of the Declaration of Helsinki and Good Clinical Practice.

### Patients

Women who were postmenopausal and over 18 years of age with histologically or cytologically confirmed, locally advanced or metastatic, ER + (HER2 −) breast cancer were eligible for enrollment in phases Ia/Ib/IIa. There were phase-specific inclusion criteria as addressed here, in Supplementary Methods, Supplementary Table S1, and Supplementary Fig. S1. Phase Ia dose escalation required ≥ 2 months since the last use of tamoxifen and ≥ 6 months since the last use of fulvestrant. Phase Ib combination treatments required no prior treatment with CDK4/6 inhibitor in cohort C1. Phase IIa dose expansion at the RP2D did not allow prior fulvestrant in cohorts A1 or B1 but allowed it in cohorts A2 and B2, and required > 2 months since the last use of tamoxifen in cohort A1.

Exclusion criteria for all three study phases are as addressed here, in Supplementary Methods, Supplementary Table S2, and Supplementary Fig. S1. Patients were excluded if they had untreated or symptomatic central nervous system metastases; endometrial disorders (history of endometrial polyps, endometrial cancer, endometrial hyperplasia, and other significant disorders); any significant cardiac dysfunction within 12 months prior to enrollment; active inflammatory bowel disease or chronic diarrhea, short bowel syndrome, or upper gastrointestinal surgery; known human immunodeficiency virus (HIV) infection; known clinically significant history of liver disease; major surgery within 4 weeks prior to enrollment; or radiation therapy within 2 weeks prior to enrollment.

### Study treatments

Patients in phase Ia dose-escalation cohorts received oral doses of GDC-0810 during 28-day cycles given once daily (QD) or twice daily (BID), with fasting and without fasting, and with a single dose given on day 7 leading into cycle 1. The starting dose in the first cohort was 100 mg per day based on preclinical studies. Dose escalation to 200 mg and by 200-mg increments in successive cohorts occurred in the absence of dose-limiting toxicities (DLTs) or conditionally in the presence of DLTs.

Phase Ib was a dose-escalation study of GDC-0810 starting at 400-mg QD, as combination treatment with 125-mg palbociclib (21 days on/7 days off) or luteinizing hormone-releasing hormone (LHRH) agonist (once every 28 days [Q4W]).

Phase IIa explored the recommended phase 2 dose (RP2D) from phase Ia. All patients received GDC-0810 600 mg under non-fasting conditions.

Patients in all three study phases continued treatment until unacceptable toxicity, disease progression, or consent withdrawal. See Supplementary Methods for other study treatment details.

### Study assessments

AEs were assessed using the National Cancer Institute-Common Terminology Criteria for Adverse Events (NCI-CTCAE v4.0). DLTs were assessed during a 35-day DLT window (7 days leading-in + 28 days in cycle 1) and included AEs related to the study drug that were grade ≥ 3 non-hematologic events (excluding alopecia), grade ≥ 3 hematologic events lasting ≥ 7 days, or AEs of any grade that led to study drug interruption for ≥ 7 days. The protocol-defined GDC-0810-specific AEs of special interest (AESI) which were non-serious adverse events that required expedited reporting to the sponsor, included DLTs occurring during the DLT assessment window, grade ≥ 2 vomiting/diarrhea, grade ≥ 3 nausea, grade ≥ 2 thromboembolic events, grade ≥ 2 vaginal or uterine hemorrhage, and grade ≥ 3 elevation of ALT or AST.

Blood samples were collected for pharmacokinetic assessments. Tumor assessments were performed at screening and every 8 weeks from cycle 1, day 1 (C1D1). Radiographic assessment of objective tumor response or disease progression was based on Response Evaluation Criteria in Solid Tumors (RECIST v1.1) [8]. Transvaginal ultrasound scans were performed to monitor endometrial thickness at screening, at every 6 months from C1D1, and at the end of treatment. Imaging with [^18^F]-fluoroestradiol positron emission tomography (FES-PET) was performed to quantify ER expression in tumors and to assess for pharmacodynamic response. Further assessment details are provided in Supplementary Methods.

### Statistical analyses

Analyses of safety, pharmacokinetics, anti-tumor activity, and data from the FES-PET imaging correlative studies were planned. Confirmatory inferential analyses and imputation for missing data were not planned due to the exploratory nature of this study. Descriptive statistics were used to summarize patient data. The number of patients to be enrolled was dependent upon the observed safety and pharmacokinetic profile during dose escalations. The safety- and efficacy-evaluable population included patients who received at least one dose of the study drug. Safety was assessed through summaries of DLTs, AEs, changes in select laboratory test results, vital signs, ECGs, and changes in endometrial thickness. Objective response (with confirmation) and clinical benefit rates (CBR) were derived per RECIST v1.1 and summarized by dose level and cohort.

## Results

### Study population

Between April 2013 and March 2020, GDC-0810 was administered to 152 female patients who were postmenopausal with ER + (HER2 −) breast cancer, as a single agent (9 dose-escalation cohorts in phase Ia [*n* = 41] and 4 dose-expansion cohorts in phase IIa [*n* = 101]) and as combination therapy (2 dose-escalation cohorts in phase Ib [*n* = 10]). Phases Ia and IIa were conducted in Spain, the Netherlands, South Korea, and the USA; phase Ib was conducted in the USA and South Korea. Patients in phase Ia dose escalation were enrolled in cohorts with or without a fasting regimen, with once or twice daily dosing (Supplementary Fig. S1). The patient population in the 3 phases was predominantly white (*n* = 127, 84%) with mean age of 60 years (range 31–79) and a high proportion with visceral disease (*n* = 88, 58%) (Table 1; Supplementary Table S3). Patients were in the metastatic disease setting. Patients in phase Ia dose escalation had received prior MBC therapy (*n* = 34, 100%) (aromatase inhibitors, tamoxifen, everolimus), chemotherapy (*n* = 29, 85%), fulvestrant (*n* = 17, 42%), and cyclin-dependent kinase (CDK 4/6) inhibitor (*n* = 1, 2%). In phase Ib combination treatments and phase IIa expansion cohorts, patients had received prior MBC therapy (*n* = 97, 87%) (aromatase inhibitors, tamoxifen, everolimus), chemotherapy (*n* = 20, 21%), fulvestrant (*n* = 22, 23%), and CDK 4/6 inhibitor (*n* = 14, 14%). In phase IIb, 105 of 111 patients had evaluable disease and 6 patients had missing or unavailable measurements.

**Table 1:** Demographics and baseline characteristics for patients in phase Ia, IIa, and Ib studies who received GDC-0810

	Phase Ia	Phase IIa				Phase Ib	
	Dose escalation	Dose expansion				Combination treatments*	
	Cohorts 1–9	A1	A2	B1	B2	C1	D1
	100–800 mg BID; 300–400 mg QD	600 mg QD	600 mg QD	600 mg QD	600 mg QD	400 mg QD + PALBO	600 mg QD + LHRH-A
	(*n* = 41)	(*n* = 19)	(*n* = 10)	(*n* = 53)	(*n* = 19)	(*n* = 4)	(*n* = 6)
Age, median years (range)	61 (33–78)	55 (35–78)	66 (47–73)	63 (41–79)	61 (37–79)	59 (42–74)	41 (31–55)
Race, *n* (%)							
Asian	2 (5)	2 (11)	1 (10)	2 (4)	0	0	3 (50)
Black or African American	1 (2)	0	0	0	2 (11)	0	0
White	36 (88)	17 (89)	8 (80)	46 (87)	15 (79)	4 (100)	1 (17)
Other	1 (2)	0	0	0	0	0	0
Missing	1 (2)	0	1 (10)	5 (9)	2 (11)	0	2 (33)
ECOG PS at baseline							
0	19 (46)	15 (79)	7 (70)	34 (65)	12 (63)	4 (100)	6 (100)
1	22 (54)	4 (21)	3 (30)	18 (35)	7 (37)	0	0
Metastatic site nos., median (range)	2 (1–9)	2 (0–4)	2 (2–4)	2 (1–6)	1 (1–5)	2 (1–2)	2 (1–5)
Presence of visceral disease, *n* (%)	26 (63)	9 (50)	9 (90)	33 (62)	8 (42)	2 (50)	1 (17)
Prior fulvestrant use, *n* (%)	17 (41)	0	7 (70)	0	15 (79)	0	0

*Combination treatment (phase Ib) included palbociclib (PALBO) (125 mg on days 1–21 of 28-day cycles) or luteinizing hormone-releasing hormone agonist (LHRH-A)

### Pharmacokinetics

The pharmacokinetic profile of GDC-0810 was linear and dose proportional up to 600 mg. GDC-0810 was rapidly absorbed with peak concentrations (median T_max_) achieved at 1–3 h after dosing. The mean terminal half-life was approximately 8 h after 600-mg QD dosing under the non-fasted condition. Minimal drug accumulation was observed following multiple dosing. At the RP2D of 600 mg QD, the average exposure of GDC-0810 at steady state was 1.5-fold (AUC_0-24 h_) to twofold (C_max_) higher when the drug was administered in non-fasted condition compared to fasted condition. Plasma exposures of glucuronide metabolites were lower than the parent GDC-0810 molecule; the average exposure of GDC-0810-N-glucuronide was approximately 3% and 10% for GDC-0810-acyl-glucuronide at steady state with 600-mg QD dosing under the non-fasted condition. The pharmacokinetic profile and parameters of GDC-0810 and its metabolites, GDC-0810-N-glucuronide and GDC-0810-acyl-glucuronide, following single and multiple doses of GDC-0810, are presented in detail elsewhere [9].

### Pharmacodynamics

**Fig. 1:**
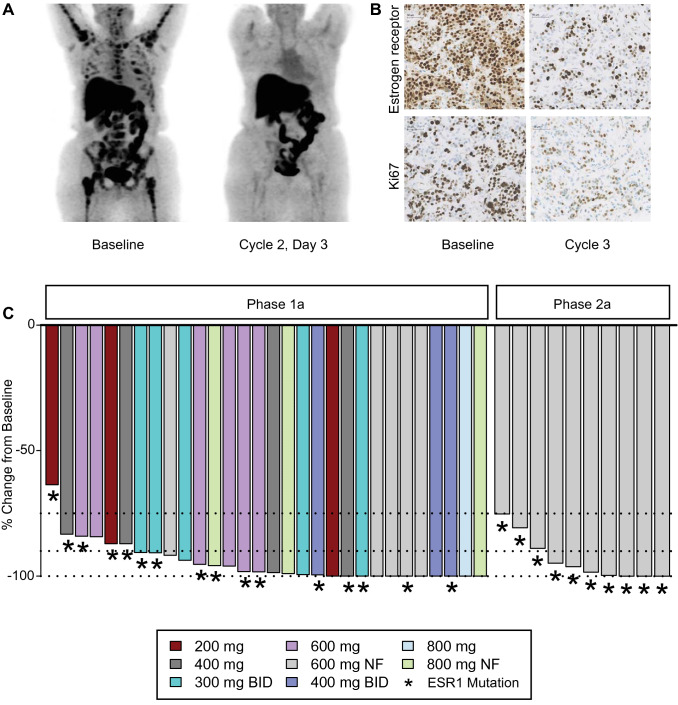
Pharmacodynamics of GDC-0810. **A** Functional imaging with ^18^F-FES-PET for patient on GDC-0810 600-mg QD (fasting regimen) at baseline and cycle 2 day 3 with best response of stable disease. **B** ER protein levels and tumor cell proliferation (Ki-67) at baseline and treatment-related reductions after 3 cycles of treatment for patient on GDC-0810 600-mg QD (non-fasting regimen) with best response of stable disease. **C** Waterfall 18F-FES-PET response for patients in six dose cohorts in phase Ia and cohort A1 in phase IIa

Pharmacodynamics analyses demonstrated robust target engagement via FES-PET imaging obtained on-treatment versus before GDC-0810 treatment (Fig. 1a), as well as in paired tumor biopsies (Fig. 1b). Overall, complete or near complete (> 90%) suppression of FES uptake was observed in 78% of all patients who had FES-PET scans (24 of 30 phase Ia and 7 of 10 phase IIa dose-expansion patients who had both baseline and C2D3 and C3D3 scans, respectively), including 18 of 26 patients with tumors harboring *ESR1* mutations (Fig. 1c). In phase Ia dose escalation, two patients had a single lesion that was considered FES-avid (SUV_max_ corrected > 1.5) at C2D3; one patient had a subsequent C3 scan which showed no avid lesions. While reduced ER and Ki-67 levels in tumor specimens were noted after treatment with GDC-0810 for one cycle (28 days of treatment) (Fig. 1b), no conclusions could be drawn because baseline and post-dose samples were only available from 3 patients.

### Study drug exposure, RP2D, and MTD

**Fig. 2:**
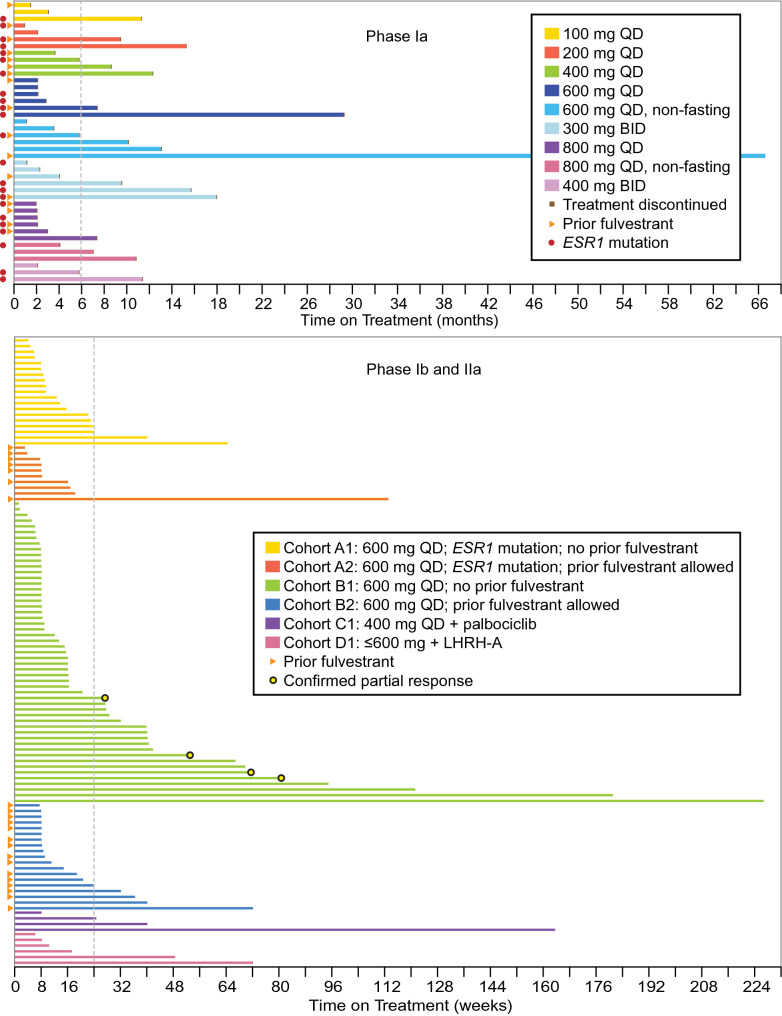
Patient time on study. Two cohorts in phase Ia, and all cohorts in phase Ib and IIa were dosed under non-fasting conditions

In phase Ia dose escalation, AEs led to dose interruption in 18 (44%) patients, dose reduction in 5 (12%), and study drug withdrawal in 2 (1%). Overall, the average treatment duration was 211 days (range 28–790) (Fig. 2). Twenty (49%) patients remained on treatment for ≥ 24 weeks. Two patients remained on study treatment for 2 years. Although a MTD was not reached in this phase Ia study, the GDC-0810 dose at 800 mg daily was considered intolerable based on the frequency of gastrointestinal AEs (nausea, vomiting, and diarrhea). The GDC-0810 dose of 600 mg QD was declared to be the RP2D when administered under fed conditions, given its overall safety, tolerability, and pharmacokinetic profile. This was the optimal biological dose (600-mg QD, fasting, and non-fasting) where patients demonstrated 80–100% response rates by FES-PET, including patients with tumors harboring *ESR1* mutations, discussed in greater detail elsewhere [10].

In phase Ib combination treatments, AEs led to dose interruption in 5 (50%) patients, dose reduction in none, and study drug withdrawal in none. The average treatment duration was 413 days (range 56–1144) and 189 days (range 43–504) for cohorts C1 and D1, respectively. Four (40%) patients remained on treatment for ≥ 24 weeks.

In phase IIa expansion cohorts, AEs led to dose interruption in 32 (32%) patients, dose reduction in 4 (4%), and study drug withdrawal in 3 (3%). The average treatment duration was 115 days (range 28–450), 142 days (range 21–791), 216 days (range 8–1586), and 129 days (range 52–504) for cohorts A1, A2, B1, and B2, respectively. Twenty-six (26%) patients remained on treatment for ≥ 24 weeks.

### Efficacy

In phase Ia dose escalation, the CBR was 39% as 16 patients had achieved clinical benefit that was defined as RECIST v1.1 responses (complete response [CR], partial response [PR], and/or stable disease [SD]) lasting for ≥ 24 weeks (6 months). For 17 patients in phase Ia who were previously treated with fulvestrant, the CBR was 35% and therefore comparable to that achieved overall (39%). For best confirmed overall outcome, 26 (63%) patients had SD, 12 (29%) had progressive disease (PD), 2 (5%) had confirmed PR, and data for one (2%) patient were missing (Table 2).

**Table 2:** Efficacy in phase Ia, IIa, and Ib studies for patients who received GDC-0810

	Phase Ia	Phase IIa				Phase Ib	
	Dose escalation	Dose expansion				Combination treatments	
	Cohorts 1–9	A1	A2	B1	B2	C1	D1
	100–800-mg BID; 300–400-mg QD	600 mg QD	600 mg QD	600 mg QD	600 mg QD	400 mg QD + PALBO	600 mg QD + LHRH-A
All patients							
Patients (*n*)	(*n* = 41)	(*n* = 19)	(*n* = 10)	(*n* = 53)	(*n* = 19)	(*n* = 4)	(*n* = 6)
Partial response	2 (5%)	0	0	4 (8%)	0	0	0
Stable disease	26 (63%)	4 (21%)	4 (40%)	17 (32%)	4 (21%)	2 (50%)	2 (33%)
Non-complete response/non-progressive disease	0	5 (26%)	0	8 (15%)	4 (21%)	1 (25%)	1 (17%)
Progressive disease	12 (29%)	9 (47%)	4 (40%)	21 (40%)	11 (58%)	1 (25%)	3 (50%)
Missing or unevaluable	1 (2%)	1 (5%)	2 (20%)	3 (6%)	0	0	0
Patients with tumors harboring *ESR1* mutations							
Patients (*n)*	(*n* = 23)	(*n* = 19)	(*n* = 9)*	(*n* = 20)	(*n* = 4)	(*n* = 0)	(*n* = 2)
Partial response	2 (9%)	0	0	1 (5%)	0	–	0
Stable disease	15 (65%)	4 (21%)	3 (33%)	8 (40%)	1 (25%)	–	1 (50%)
Non-complete response/non-progressive disease	0	5 (26%)	0	3 (15%)	1 (25%)	–	0
Progressive disease	6 (26%)	9 (47%)	4 (44%)	7 (35%)	2 (50%)	–	1 (50%)
Missing or unevaluable	0	1 (5%)	2 (22%)	1 (5%)	0	–	0

*Results for one patient who was enrolled in cohort A2 based on local determination of tumor harboring *ESR1* mutation could not be confirmed due to insufficient tumor tissue for biopsy; this patient was excluded from the confirmed *ESR1* mutation group

In phases Ib combination treatments and IIa expansion cohorts (*n* = 111), the CBR was 22% (*n* = 24). The best confirmed overall outcome in phases Ib and IIa included 4 (8%) PR, 33 (30%) SD, 19 (17%) non-complete response or non-progressive disease (non CR/PD), 49 (44%) PD, and 6 (5%) missing or unevaluable (Table 2).

Overall, in the 3 study phases, there were 6 (4%) partial responses, 59 (39%) stable disease, 19 (13%) non-complete response/non-progressive disease, 61 (40%) progressive disease, 5% missing/unevaluable, and the CBR was 26% (*n* = 40). Patients with clinical benefit did not have significantly different uptake of FES at baseline or a greater reduction in FES uptake on treatment than patients without clinical benefit and a greater reduction in uptake was not associated with a longer time on study.

### Efficacy in patients with tumors harboring *ESR1* mutations

**Fig. 3:**
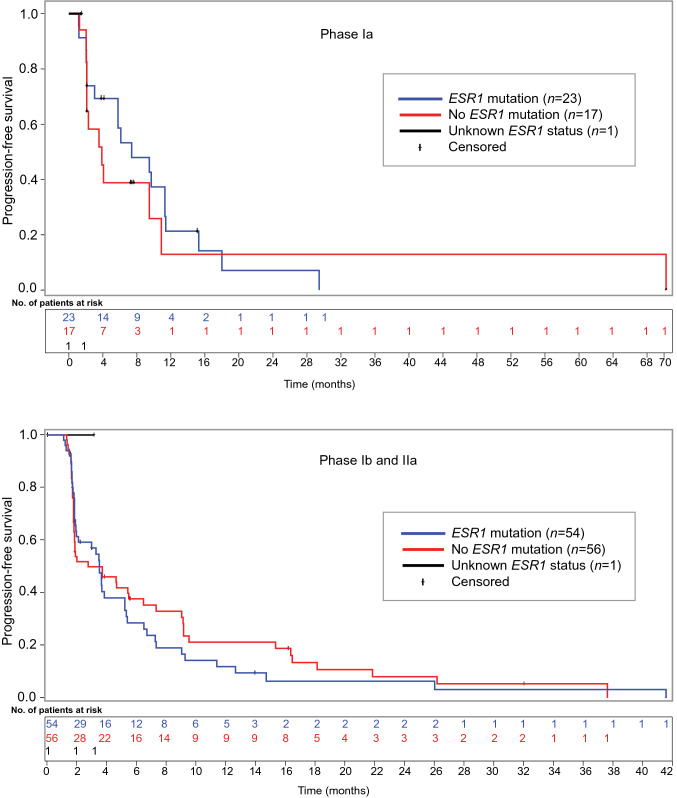
Kaplan–Meier plot of progression-free survival for patients in phase Ia (top) and phases 1b/IIa (bottom)

Among 152 patients with ER + (HER2 −) MBC in the three study phases, 59 (39%) had an outcome of stable disease; data cutoff was April 2016 for phase Ia and March 2020 for phases Ib and IIa. In comparison, among 77 patients with tumors harboring *ESR1* mutations, 32 (42%) patients had an outcome of stable disease (Table 2). In patients (*n* = 77) with tumors harboring *ESR1* mutations, there were 3 (4%) partial responses, 32 (42%) stable disease, 9 (12%) non-complete response/non-progressive disease, 29 (38%) progressive disease, and 4 (5%) missing/unevaluable (Table 2; Fig. 3). For patients (*n* = 77) with tumors harboring *ESR1* mutations, the CBR in phase Ia (10 of 23 patients) was 57% and 15% in phase Ib/IIa (8 of 54 patients).

For the 111 patients in study phases Ib/IIa, the median time to event was 3.5 months (95% confidence interval (*CI* 1.9, 5.2). In comparison, for the 54 patients in study phases Ib/IIa with tumors harboring *ESR1* mutations, the median time to event was the same – 3.5 months (*CI* 1.9, 5.2) (Fig. 3).

### Safety

All patients who received the study drug experienced ≥ 1 AE regardless of causality (Table 3). The most common AEs regardless of attribution across the 3 phases *N* = 152) in ≥ 25% of patients included diarrhea (*n* = 95, 63%), nausea (*n* = 82, 54%), fatigue (*n* = 77, 51%), vomiting (*n* = 47, 31%), constipation (*n* = 42, 28%), and decreased appetite (*n* = 38, 25%).

**Table 3:** Adverse events in phase Ia, IIa, and Ib by preferred term for events with occurrence in > 25% of total population for all grades and grades 3–5

	Phase Ia	Phase IIa				Phase Ib		
	Dose escalation	Dose expansion				Combination treatments		All 3 study phases
	Cohorts 1–9	A1	A2	B1	B2	C1	D1	
	100–800-mg BID; 300–400-mg QD	600 mg QD	600 mg QD	600 mg QD	600 mg QD	400 mg QD + palbociclib	≤ 600 mg QD + LHRH agonist	
	(*n* = 41)	(*n* = 19)	(*n* = 10)	(*n* = 53)	(*n* = 19)	(*n* = 4)	(*n* = 6)	(*N* = 152)
Diarrhea	32 (78)	10 (53)	5 (50)	29 (55)	11 (58)	2 (50)	6 (100)	95 (63)
Grades 3–5	0	0	1 (10)	5 (9)	0	0	1 (17)	7 (5)
Nausea	28 (68)	8 (42)	6 (60)	24 (45)	9 (47)	3 (75)	4 (67)	82 (54)
Grades 3–5	1 (2)	0	0	0	0	0	0	1 (1)
Fatigue	28 (68)	8 (42)	2 (20)	27 (51)	7 (37)	2 (50)	3 (50)	77 (51)
Grades 3–5	3 (7)	0	0	2 (4)	0	1 (25)	0	6 (4)
Vomiting	16 (39)	8 (42)	4 (40)	14 (26)	2 (11)	0	3 (50)	47 (31)
Grades 3–5	1 (2)	2 (11)	0	1 (2)	0	0	0	4 (3)
Constipation	17 (42)	2 (11)	0	16 (30)	4 (21)	0	3 (50)	42 (28)
Grades 3–5	0	0	0	1 (2)	0	0	0	1 (1)
Decreased appetite	13 (32)	6 (32)	3 (30)	9 (17)	2 (11)	1 (25)	4 (67)	38 (25)
Grades 3–5	0	0	0	0	0	0	0	0
Anemia	18 (44)	2 (11)	2 (20)	6 (11)	4 (21)	2 (50)	0	34 (22)
Grades 3–5	1 (2)	0	1 (10)	1 (2)	1 (5)	0	0	4 (3)
Arthralgia	12 (29)	4 (21)	0	14 (26)	0	1 (25)	1 (17)	32 (21)
Grades 3–5	0	0	0	0	0	0	0	0
Cough	13 (32)	2 (11)	1 (10)	8 (15)	4 (21)	1 (25)	1 (17)	30 (20)
Grades 3–5	0	0	0	1 (2)	0	0	0	1 (1)
Dyspepsia	11 (27)	6 (31)	2 (20)	6 (11)	2 (11)	0	3 (50)	30 (20)
Grades 3–5	0	0	1 (10)	0	0	0	0	1 (1)
Flatulence	11 (27)	4 (21)	0	13 (25)	0	0	1 (17)	29 (19)
Grades 3–5	0	0	0	0	0	0	0	0
Abdominal pain	12 (29)	3 (16)	0	10 (19)	1 (5)	0	2 (33)	28 (18)
Grades 3–5	1 (2)	1 (5)	0	1 (2)	0	0	0	3 (2)
AST increased	17 (42)	1 (5)	2 (20)	4 (8)	1 (5)	1 (25)	0	26 (17)
Grades 3–5	2 (5)	0	0	2 (4)	0	0	0	4 (3)

During dose escalation, 40 (98%) patients in phase Ia experienced ≥ 1 AE considered related to GDC-0810 by the investigator (Supplementary Table S4). Fifteen (37%) patients in phase Ia experienced AESIs considered related to GDC-0810 by the investigator, including grade ≥ 2 diarrhea (*n* = 14, 34%), grade ≥ 2 vomiting (*n* = 7, 17%), grade ≥ 2 thromboembolic event (*n* = 3, 7%), grade ≥ 3 nausea (*n* = 1, 2%), and a DLT (diarrhea, 800-mg QD). Thirteen (32%) patients experienced ≥ 1 serious AEs (SAEs) regardless of causality; one of the 20 SAEs was considered related to the study drug by the investigator (Supplementary Table S5) consisting of grade 4 pulmonary embolism.

In phases Ib/IIa, one hundred and one (91%) patients experienced ≥ 1 AE of any grade considered by the investigators to be related to GDC-0810. Diarrhea was the most commonly occurring grade ≥ 3 event (*n* = 37, 33%). Fifty-five (50%) patients experienced AESIs that included events within the MedDRA SOC of reproductive/breast event (*n* = 29, 26%), grade ≥ 2 diarrhea (*n* = 25, 23%), grade ≥ 2 vomiting (*n* = 9, 8%), grade ≥ 2 thromboembolic event (*n* = 7, 6%), and grade ≥ 3 elevation of ALT/AST (*n* = 2, 2%). Three (3%) patients experienced grade 5 AEs including progression of breast cancer (*n* = 2) and acute renal injury (*n* = 1); the 3 events were considered unrelated to GDC-0810 by the investigators.

Across all 3 phases, there were 33 patients who reported SAEs regardless of attribution in phase 1a (*n* = 13), 1b (*n* = 3), and 2a (*n* = 17) studies (Supplementary Tables S5 and S6). Six (15%) deaths were reported in phase Ia study, attributed to disease progression (*n* = 3) and grade 5 SAEs (*n* = 3), none related to the study treatment. There were no deaths reported in phase Ib and 3 deaths in phase IIa due to progression of the grade 5 AEs described above (breast cancer [*n* = 2] and acute renal injury [*n* = 1]).

Among 10 patients in phase Ia dose escalation with a baseline and at least one follow-up scan, 9 (90%) patients showed an increase in the thickness of endometrium compared to baseline, with a median change in thickness of 3.5 mm (range 0 to 11) and mean change of 4.4 mm. There were no treatment discontinuations due to AEs of vagina bleeding or endometrial cancer; a grade 1 vagina hemorrhage in one patient was considered unrelated to GDC-0810 following an endometrial biopsy. In phase Ib combination treatments and IIa dose-expansion studies, 28 of 36 (78%) patients for whom baseline and post-baseline scans were available showed thickening of the endometrium.

## Discussion

This proof-of-concept study demonstrated that GDC-0810 was safe and generally well tolerated with predictable pharmacokinetics, evidence of robust target engagement, and encouraging anti-tumor activity in heavily pretreated patients with advanced or metastatic ER + (HER2 −) breast cancer. Most of the AEs were grade 1–2 and manageable with dose interruption, medication, and/or supportive care, with diarrhea being the most common AE with an average onset timeline of 29 days in the phase Ia dose-escalation cohorts. The MTD was not reached; there was one DLT during the DLT window (day 7 to end of cycle 1; 35 days total). Based on safety, pharmacokinetic, and pharmacodynamics data, 600-mg QD was the RP2D for GDC-0810 when administered under non-fasted conditions.

GDC-0810 is a SERD therapy in a landscape where fulvestrant is the only approved first-generation SERD, most frequently used in the second-line setting for ER + advanced or MBC. Combination with CDK4/6 inhibitors has shown a clear benefit over fulvestrant monotherapy in patients who progress on prior endocrine therapy. While fulvestrant has been and continues to be highly impactful in the MBC disease setting, its disadvantages include poor solubility and pharmacokinetic properties requiring intramuscular injections and limited activity in patients with tumors harboring *ESR1* mutations [11]. In this study, GDC-0810 demonstrated activity as a single agent including in patients with tumors harboring *ESR1* mutations. Furthermore, the clinical benefit rate for GDC-0810 was the same regardless of whether patients received or did not receive prior fulvestrant. In the phase III EMERALD trial investigating elacestrant (a SERD) in patients with MBC who received endocrine + CDK4/6 previously, the PFS was 2.8 months with elacestrant and 1.9 months with standard-of-care (SOC) fulvestrant or an aromatase inhibitor; patients with tumors harboring *ESR1* mutations had a median PFS of 3.8 months with elacestrant and 1.9 months with SOC [12, 13]. In the current study evaluating GDC-0810, the median PFS for phase Ib/IIa was 3.5 months (combination treatments in phase Ib; dose expansion at RP2D in phase IIa), and this was the same for patients with or without *ESR1* mutations in tumors.

We evaluated combination treatments of GDC-0810 with palbociclib or a LHRH agonist in phase Ib cohorts. Previously, LHRH agonists have been combined with endocrine therapies without significantly affecting their safety profiles in adjuvant and metastatic disease settings for women who were peri- and premenopausal [14, 15]. Guidelines for women in these groups who have advanced breast cancer suggest treatment with ovarian suppression; women are rendered postmenopausal with LHRH agonists and are treated with therapies for the postmenopausal setting. In the current study, patients were postmenopausal without any perceived benefit from LHRH therapy. However, the safety profile of the combination treatment in this study relates to the peri- or premenopausal population who become postmenopausal following LHRH therapy. We found AEs observed for GDC-0810 in combination treatment with palbociclib or LHRH agonist to be similar to those for other endocrine agents; the AEs were amenable to monitoring, manageable, and reversible. Importantly, the benefit from endocrine therapies is expected to be similar for the different patient populations. For example, in the PALOMA-3 study, ~ 80% of the patients were postmenopausal and ~ 20% were peri- or premenopausal; both populations demonstrated similar benefit to combination treatment of a SERD (fulvestrant) and palbociclib, while including LHRH therapy for the latter group [15].

In a patient population with advanced or MBC, FES-PET is a validated method for localizing ER-expressing tumors and has also been reported to predict response to endocrine therapy [16–18]. Activity resulting from ER-targeting agents manifests as a decline in FES uptake post-treatment in comparison to baseline and is indicative of ER engagement, although not necessarily of ER degradation. In patients receiving fulvestrant, FES-PET has been used to demonstrate residual ER activity in 38% of patients, thereby signifying inadequate dosing of fulvestrant for targeting the available tumor ERs, which has been associated with early progression [16]. In the current study, ≥ 80% of patients who received GDC-0810 at 600 -g QD (fasting and non-fasting) demonstrated complete or near-complete reduction in FES uptake, including in patients with tumors harboring *ESR1* mutations. There was no correlation between FES reduction and clinical benefit in this study, indicating that while high target occupancy may be necessary for ER-targeting agents, achieving clinical benefit and disease control depends on additional factors, such as ER dependency of the tumor.

Breast cancer patients treated with tamoxifen, a selective ER modulator (SERM), have shown clinically significant effects on endometrial thickening. ER is a ligand-inducible transcription factor that contains a central DNA-binding domain; SERMs can have antagonist activity on one domain with agonist activity on another [19]. While highly effective for breast cancer, tamoxifen exhibits ER agonist activity in the uterus and is associated with an increased risk of endometrial hyperplasia and malignancy [20]. Endometrial safety is therefore an important consideration in the development of endocrine agents. In contrast to tamoxifen, fulvestrant is not associated with endometrial thickening. GDC-0810 is a SERD with similarity to fulvestrant, known to suppress ER transcriptional activity by slowing the intra-nuclear mobility of ER [19]. However, endometrial thickening was observed in this study, similar to the class effects of SERM ER agonist activity in the uterus, although no ensuing AEs resulted in treatment discontinuations. The short exposure duration in this study was not expected to result in the development of endometrial cancer. Interestingly, aromatase inhibitors consecutive to or in combination with SERM have been shown to ameliorate the SERM effects of endometrial thickening [21] and could potentially be part of a combination regimen with SERDs. There were no clinically significant ER agonist effects on the lipid profiles of patients treated with GDC-0810.

While the sponsor decision has been to discontinue GDC-0810 development due to an inferior risk/benefit profile relative to other oral SERDs demonstrating early evidence of full ER antagonism and improved tolerability [22, 23], this study has provided important clinical data for continued development of the SERD landscape, including FES-PET utility in the characterization of ER-expressing tumors, activity of this SERD in patients with tumors harboring *ESR1* mutations, and the overall clinical safety and pharmacokinetic profile for a SERD. GDC-0810 is the first molecule to be prospectively optimized for ER degradation [6, 7]. This study has contributed to research toward a newer next-generation oral SERDs (GDC-0927 and giredestrant) with improved ER antagonism, no uterine agonism, better toxicity profile, and superior efficacy profile as single agent and combination [22, 24–26].

## Supplementary Information

Below is the link to the electronic supplementary material.

Supplementary file1 (PDF 455 kb)

## Data Availability

Qualified researchers may request access to individual patient-level data through the clinical study data request platform (https://vivli.org/). Other details on Roche’s criteria for eligible studies are available here (https://vivli.org/members/ourmembers/). For further details on Roche’s Global Policy on the Sharing of Clinical Information and how to request access to related clinical study documents, see here (https://www.roche.com/innovation/process/clinical-trials/data-sharing).
