# Supplementary material for: The oral selective estrogen receptor degrader GDC-0810 (ARN-810) in postmenopausal women with hormone receptor-positive HER2-negative (HR + /HER2 −) advanced/metastatic breast cancer
Source: Breast Cancer Res Treat. 2022 Nov 19;197(2):319–31. doi: 10.1007/s10549-022-06797-9 (PMC9823088; doi:10.1007/s10549-022-06797-9)
Supplement: Supplementary file 1 — Supplementary file1 (PDF 455 kb) [file 10549_2022_6797_MOESM1_ESM.pdf]

The oral selective estrogen receptor degrader GDC-0810 (ARN-810) in postmenopausal women with hormone receptor-positive HER2-negative (HR+/HER2−) advanced/metastatic breast cancer

Aditya Bardia[1], Ingrid Mayer[2, 3], Eric Winer[4, 5], Hannah M. Linden[6], Cynthia X. Ma[7], Barbara A. Parker[8], Meritxell Bellet[9], Carlos L. Arteaga[10], Sravanthi Cheeti[11], Mary Gates[11], Ching-Wei Chang[11], Jill Fredrickson[11], Jill M. Spoerke[11], Heather M. Moore[11], Jennifer Giltnane[11], Lori S. Friedman[11, 12], Edna Chow Maneval[12], Iris Chan[11], and Komal Jhaveri[13]

1 Massachusetts General Hospital Cancer Center, Harvard Medical School, Boston, MA
2 Vanderbilt-Ingram Cancer Center, Nashville, TN<br>3 AstraZeneca, Gaithersburg, MD
4 Dana-Farber Cancer Institute, Boston, MA<br>5 Yale Cancer Center, New Haven, CT
6 University of Washington, Seattle, WA
7 Washington University School of Medicine, St. Louis, MO
8 University of California San Diego Moores Cancer Center, San Diego, CA
9 Vall d'Hebron Institute of Oncology, Barcelona, Spain
10 UT Southwestern Simmons Comprehensive Cancer Center, Dallas, TX
11 Genentech, Inc., South San Francisco, CA
12 ORIC Pharmaceuticals, South San Francisco, CA
13 Memorial Sloan Kettering Cancer Center, New York, NY, Weill Cornell Medical College, New York, NY

Supplementary Methods

Patients

Women who were postmenopausal and over 18 years of age with histologically or cytologically confirmed, locally advanced or metastatic, ER+ (HER2−) breast cancer were eligible for enrollment in phases Ia/Ib/IIa. There were phase-specific inclusion criteria as addressed here and in Supplementary Table S1. Phase Ia required ≥2 months since the last use of tamoxifen and ≥6 months since the last use of fulvestrant. Phase Ib required no prior treatment with CDK4/6 inhibitor in cohort C1. Phase IIa did not allow prior fulvestrant in cohorts A1 or B1 but allowed it in cohorts A2 and B2, and required >2 months since the last use of tamoxifen in cohort A1. Inclusion criteria specific to phase IIa, cohorts A1 and A2, included the presence of measurable disease as per RECIST v1.1 and centrally confirmed *ESR1* mutations in the baseline plasma circulating tumor DNA (ctDNA). For phase IIa, cohorts B1 and B2, there was a requirement for patients with measurable disease or evaluable bone disease, and disease progression following ≤1 prior treatment with an aromatase inhibitor (AI) in the advanced/metastatic disease setting (relapse ≥12 months from completion of adjuvant treatment or progression ≥6 months after treatment in the advanced/metastatic disease setting).

Other inclusion criteria for all three study phases were ≥2 weeks since the last use of any other anti-cancer hormonal therapy; ≥3 weeks since the last use of any chemotherapy; Eastern Cooperative Oncology Group (ECOG) performance status ≤2, for phase Ia, and 0–1 for phases Ib and IIa; resolution of all acute toxic effects of prior therapy or surgical procedures to baseline or grade ≤1; and adequate organ function. Exclusion criteria for all three study phases are as addressed here and in Supplementary Table S2. Patients were excluded if they had untreated or symptomatic central nervous system metastases; endometrial disorders (history of endometrial

polyps, endometrial cancer, endometrial hyperplasia, and other significant disorders); any significant cardiac dysfunction within 12 months prior to enrollment; active inflammatory bowel disease or chronic diarrhea, short bowel syndrome, or upper gastrointestinal surgery; known human immunodeficiency virus (HIV) infection; known clinically significant history of liver disease; major surgery within 4 weeks prior to enrollment; or radiation therapy within 2 weeks prior to enrollment.

There were phase-specific exclusion criteria as well. In phase Ib, cohort C1 excluded patients with a history of venous thromboembolic event requiring therapeutic anticoagulation or vaginal bleeding within 2 months prior to enrollment. In phase IIa, cohorts A1 and A2 excluded patients with >1 chemotherapy in the advanced/metastatic disease setting. In phase IIa, cohort B1 excluded patients with >1 prior AI in the advanced/metastatic disease setting or prior chemotherapy except as adjuvant prior to ≥12 months. In phase IIa, cohort B2 excluded patients with >1 prior AI or >1 prior chemotherapy in the advanced/metastatic disease setting.

Study treatments

Patients in phase Ia in dose escalation received oral doses of GDC-0810 during 28-day cycles given once daily (QD) or twice-daily (BID), with fasting and without fasting, and with a single dose given on day −7 leading into cycle 1. The starting dose in the first cohort was 100 mg per day based on preclinical studies. Dose escalation to 200 mg and by 200 mg increments in successive cohorts occurred in the absence of dose-limiting toxicities (DLTs), or conditionally in the presence of DLTs. A DLT was defined as any adverse event (AE) occurring during the DLT window deemed by the investigator or sponsor to be related to the study drug unless they were clearly related to disease progression or other cause; DLT incidences were evaluated from day −7 through the first cycle (28 days) of treatment for a total of 35 days. If one of the first 3

patients in a dose cohort experienced a DLT, 3 additional patients were assigned to that dose; dose escalation to the next level proceeded if DLTs were experienced by ≤1 patient out of 6. If ≥2 patients experienced DLTs at a given dose, a lower dose was to be declared the maximum tolerated dose (MTD).

Phase Ib was a dose-escalation study of GDC-0810 starting at 400 mg QD, as combination treatment with 125 mg palbociclib (21 days on/7 days off) or luteinizing hormone releasing hormone (LHRH) agonist (once every 28 days [Q4W]).

Phase IIa explored the recommended phase 2 dose (RP2D) from phase Ia. All patients received GDC-0810 600 mg under non-fasting conditions. Cohorts A1 and A2 enrolled patients with tumors harboring *ESR1* mutations with no prior fulvestrant use in cohort A1 while prior fulvestrant use was allowed in cohort A2. Cohorts B1 and B2 enrolled patients whose disease had progressed and who may have had one or no AI therapy; there was no prior fulvestrant use in cohort B1 while prior fulvestrant use was allowed in cohort B2.

Patients in all three study phases continued treatment until unacceptable toxicity, disease progression, or consent withdrawal. No more than two dose reductions were allowed. For any patient with a DLT in phase Ia, treatment was withheld until the toxicity resolved to grade ≤1 or baseline; treatment could resume at the next lower dose if deemed clinically beneficial. For treatment interruptions other than due to DLTs, dosing was resumed at the discretion of the investigator when toxicity resolved to grade ≤1 or baseline.

For AEs related to the study drug, patients were withdrawn from study treatment for dose interruptions lasting longer than 28 days. For missed doses, the next scheduled dose of GDC-0810 was administered without compensating for the missed one. For patients with a clinical need for a particular CYP2B6 or CYP2C substrate drug, dose adjustments and/or use of

alternative medications were recommended due to the inhibitory effects of GDC-810 on CYP2B6 and CYP2C enzymes. No hormonal therapy, chemotherapy, immunotherapy, or experimental anti-cancer medications were permitted during the study.

Study assessments

Blood samples were collected for pharmacokinetic assessment on lead-in day −7 and cycle 2 day 1 (C2D1) at predose (0 hour) and postdose (0.5, 1, 1.5, 2, 3, 4, 6, 8, and 24 hours), (plus at 48 hours postdose for day−7). Full plasma pharmacokinetic profiles were obtained for GDC-0810 and its glucuronide metabolites (acyl-glucuronide and N-glucuronide) using a validated liquid chromatography/ tandem mass spectrometry method, and analyzed using non-compartmental methods for all patients in the dose escalation cohorts.

Tumor assessments were performed at screening and every 8 weeks from cycle-1, day-1 (C1D1). Radiographic assessment of objective tumor response or disease progression was based on Response Evaluation Criteria in Solid Tumors (RECIST v1.1). Transvaginal ultrasound scans were performed to monitor endometrial thickness at screening, at every 6 months from C1D1, and at the end of treatment. Imaging with [^{18}F]-fluoroestradiol positron emission tomography (FES-PET) was performed to quantify ER expression in tumors and to assess for pharmacodynamic response. In phase Ia, FES-PET scans were acquired at screening and on C2D3 at 18-24 hours post the C2D2 dose. Scans were repeated during cycle 3 if the C2D3 scan showed incomplete pharmacodynamic activity. In phase IIa cohort A1 patients, FES-PET scans were acquired at screening and on C3D3 at 18-24 hours post the C3D2 dose. Scans were repeated at the time of progression if feasible. The mean percent change from baseline in SUV_{max} (corrected for background activity) for up to five index lesions was calculated for each patient; more details are presented in (1). Optional tumor biopsies were collected at baseline (screening)

and after 4 weeks of treatment (C2D8 ± 1 day, during the period between ≥ 2 and ≤ 12 hours postdose) for immunohistochemistry and DNA/RNA analyses. Serial biopsies from the same tissue/lesion were collected when feasible. *ESR1* mutations in circulating tumor DNA were determined from baseline plasma collections using the Sysmex Inostics (Baltimore, Maryland) BEAMing digital PCR assay, defined as nucleotide substitutions that result in the following amino acid changes: E380Q, S463P, V534E, P535H, L536H/P/Q/R, Y537N/S/C, and D538G.

Supplementary Results

Safety

All patients who received the study drug experienced ≥1 AE regardless of causality (Table 3). The most common AEs regardless of attribution across phases Ia/Ib/IIa (*N*=152) in ≥25% of patients overall included diarrhea (*n*=95, 63%), nausea (*n*=82, 54%), fatigue (*n*=77, 51%), vomiting (*n*=47, 31%), constipation (*n*=42, 28%), and decreased appetite (*n*=38, 25%). During dose escalation, 40 (98%) patients in phase Ia experienced ≥1 AE related to GDC-0810, the most common of which were diarrhea (*n*=32, 78%), fatigue (*n*=28, 68%), nausea (*n*=28, 68%), anemia ((*n*=18, 44%), AST increased (*n*=17, 42%), constipation (*n*=17, 42%), and vomiting (*n*=16, 39%) (Supplementary Table S4). Fifteen (37%) patients in phase Ia experienced AESIs related to GDC-0810, including grade ≥2 diarrhea (*n*=14, 34%), grade ≥2 vomiting (*n*=7, 17%), grade ≥2 thromboembolic event (*n*=3, 7%), grade ≥3 nausea (n=1, 2%), and a DLT (diarrhea, 800 mg QD). The mean time to onset for any grade diarrhea in phase Ia was 29 days (range: 1-176 days). Thirteen (32%) patients experienced ≥1 serious AEs (SAEs) regardless of causality; one of the

20 SAEs was related to the study drug (Supplementary Table S5). One (2%) patient experienced one SAE reported as related to GDC-0810 consisting of grade 4 pulmonary embolism. Pneumonia and fracture were reported by two (5%) patients each and were the only SAEs reported in more than one patient. Overall, 205 (49%) patients in phase Ia experienced grade ≥3 AEs regardless of causality, including 3 (7%) patients with grade ≥3 AEs related to GDC-0810 which were a grade 4 pulmonary embolism (which was an SAE) and two grade 3 diarrhea. Apart from the pulmonary embolism, two other patients experienced grade ≥2 thromboembolic events; all patients recovered and the AEs/SAE resolved.

In phases Ib/IIa, the most common AEs were diarrhea (*n*=63, 57%), nausea (*n*=54, 49%), fatigue (*n*=49, 44%), and vomiting (*n*=31,28%). One hundred and one (91%) patients experienced ≥1 AE of any grade considered by the investigators to be related to GDC-0810, the most common of which were diarrhea (*n*=50, 45%), nausea (*n*=44, 40%), and fatigue (*n*=42, 38%). Diarrhea was the most commonly occurring grade ≥3 event (*n*=37, 33%). Fifty-five (50%) patients experienced AESIs that included reproductive/breast event (*n*=29, 26%), grade ≥2 diarrhea (*n*=25, 23%), grade ≥2 vomiting (*n*=9, 8%), grade ≥2 thromboembolic event (*n*=7, 6%), and grade ≥3 elevation of ALT/AST (*n*=2, 2%). Three (3%) patient experienced grade 5 AEs include progression of breast cancer in two patients and acute renal injury in the third; the 3 events were considered unrelated to GDC-0810 by the investigators.

Across all 3 phases, there were 33 patients who reported SAEs regardless of attribution in phase 1a (*n*=13), 1b (*n*=3), and 2a (*n*=17) studies (Supplementary Tables S5 and S6). Six (15%) deaths were reported in phase Ia study, attributed to disease progression (*n*=3) and grade 5 SAEs (*n*=3), none related to the study treatment. There were no deaths reported in phase Ib and 3 deaths in phase IIa due to progression of breast cancer (*n*=2) and acute kidney disease (*n*=1).

To assess for any potential effect of GDC-0810 on the uterus, transvaginal ultrasound scans were performed. Among 10 patients in phase Ia with a baseline and at least one follow-up scan, 9 (90%) patients showed an increase in the thickness of endometrium compared to baseline, with a median change in thickness of 3.5 mm (range 0 to 11) and mean change of 4.4 mm. There were no treatment discontinuations due to AEs of vagina bleeding or endometrial cancer; a grade 1 vagina hemorrhage in one patient was considered unrelated to GDC-0810 following an endometrial biopsy. In phase Ib and IIa studies, 28 of 36 (78%) patients for whom baseline and post-baseline scans were available showed thickening of the endometrium.

REFERENCES

1. Wang Y, Ayres KL, Goldman DA, Dickler MN, Bardia A, Mayer IA, et al. (18)F-Fluoroestradiol PET/CT Measurement of Estrogen Receptor Suppression during a Phase I Trial of the Novel Estrogen Receptor-Targeted Therapeutic GDC-0810: Using an Imaging Biomarker to Guide Drug Dosage in Subsequent Trials. Clin Cancer Res. 2017;23:3053-60.

Supplementary Tables

Supplementary Table S1. Inclusion criteria

**Inclusion criteria common to all 3 study phases:**
○ Histologically or cytologically proven diagnosis of adenocarcinoma of the breast with evidence of either locally recurrent disease not amenable to resection or radiation therapy with curative intent, or metastatic disease, both progressing after at least 6 months of endocrine therapy for ER+ breast cancer.
○ ER-positive tumor (staining in ≥1% cells by immunohistochemistry [IHC]).
○ HER2-negative tumor (IHC staining or fluorescence in situ hybridization [FISH]).
○ At least 3 weeks since last use of any chemotherapy.
○ Postmenopausal females, 18 years of age or older.
○ Resolution of all acute toxic effects of prior therapy/surgical procedures to baseline or Grade ≤1 (except alopecia or other toxicities not considered a safety risk for the patient).
○ Adequate organ function (hematological, liver, kidney).
○ Signed and dated informed consent documented prior to enrollment.
○ Birth control methods in use for women of childbearing potential.

**Inclusion criteria specific to phase Ia:**	**Inclusion criteria specific to phase Ib:**	**Inclusion criteria specific to phase IIa:**
o ECOG performance status ≤2.<br>o At least 2 months since last use of tamoxifen.<br>o At least 6 months since last use of fulvestrant.<br>o At least 2 weeks since last use of any other anti-cancer hormonal therapy.	o ECOG performance status <2.<br>o Documented sensitivity to prior hormonal therapy.<br>o At least 2 weeks since last use of any anti-cancer hormonal therapy.<br>Cohort C1:<br>o No prior CDK4/6.<br>Cohort D1:<br>o No prior LHRH agonist.	o ECOG performance status 0 or 1.<br>o At least 2 weeks since last endocrine therapy.<br>Cohort A:<br>o Confirmed *ESR1* mutations of the LBD.<br>o Evaluable disease (RECIST Version 1.1).<br>o Cohort A1: No prior fulvestrant and at least 2 months since last use of tamoxifen.<br>o Cohort A2: Prior fulvestrant allowed.<br>Cohort B:<br>o Disease progression after ≤ 1 prior treatment with AI in advanced/metastatic setting.<br>o Measurable disease/evaluable bone disease<br>o Cohort B1: No prior fulvestrant.<br>o Cohort B2: Prior fulvestrant allowed.

Supplementary Table S2. Exclusion criteria

**Exclusion criteria common to all 3 study phases:**
o Untreated or symptomatic CNS metastases.
o A history of endometrial polyps, endometrial cancer, atypical endometrial hyperplasia, or other significant endometrial disorders.
o Current treatment with any systemic anti-cancer therapies for advanced disease or any systemic experimental treatment on another clinical trial.
o Diagnosis of any secondary malignancy within 2 years prior to enrollment, except for adequately treated basal cell or squamous cell skin cancer, or carcinoma in situ.
o Any of the following within 12 months prior to enrollment: myocardial infarction, severe/unstable angina, ongoing cardiac dysrhythmias of Grade ≥ 2, atrial fibrillation of any grade, coronary/peripheral artery bypass graft, symptomatic congestive heart failure, or cerebrovascular accident including transient ischemic attack.
o Active inflammatory bowel disease or chronic diarrhea, short bowel syndrome, or upper gastrointestinal surgery/gastric resection.
o Known human immunodeficiency virus (HIV) infection.
o Known clinically significant history of liver disease consistent with Child-Pugh Class B or C, including active viral or other hepatitis, current alcohol abuse, or cirrhosis.
o Major surgery within 4 weeks prior to enrollment or radiation therapy within 2 weeks prior to enrollment.
o Other severe acute or chronic medical or psychiatric condition or laboratory abnormality that may increase the risk associated with study participation.

Exclusion criteria specific to phase Ia	Exclusion criteria specific to phase Ib	Exclusion criteria specific to phase IIa
o More than two prior chemotherapies in the advanced/metastatic setting.	o More than two prior chemotherapies including prior adjuvant chemotherapy.<br><br>o Cohort C1: History of venous thromboembolic event requiring therapeutic anticoagulation; vaginal bleeding within prior 2 months.	o Cohort A1: Prior fulvestrant,<br><br>o Cohort A1 & A2: More than1 chemo-therapy in advanced/metastatic setting.<br><br>o Cohort B1: Prior fulvestrant.<br><br>o Prior chemotherapy in the advanced/ metastatic setting.<br><br>o Cohort B2: More than 1 prior chemo-therapy in the advanced/metastatic setting.<br><br>o Cohort B1 & B2: More than 1 prior AI in the advanced/metastatic setting.

Supplementary Table S3. Demographics and baseline characteristics of the study population in phase 1a. All cohorts except two received GDC-0810 in the fasting state

	100 mg QD (*n*= 3)	200 mg QD (*n*= 4)	400 mg QD (*n*= 4)	600 mg QD (*n*= 6)	600 mg QD (*n*=6) **Non-Fasting**	300 mg **BID** (*n*=6)	800 mg QD (*n*=6)	800 mg QD (*n*=3) **Non-Fasting**	400 mg **BID** (*n*=3)	All Patients (*N*=41)
Age, median years (range)	68 (60-71)	63 (53-75)	59 (55-70)	56 (43-67)	71 (57-78)	59 (35-69)	51 (33-66)	58 (55-62)	67 (60-67)	61 (33-78)
Race, *n* (%)										
Asian	0	0	0	0	1 (17)	1 (17)	0	0	0	2 (5)
Black or African American	0	0	1 (17)	0	0	0	0	0	0	1 (2)
White	2 (67)	4 (100)	3 (75)	6 (100)	5 (83)	4 (67)	6 (100)	3 (100)	3 (100)	36 (88)
Other	1 (33)	0	0	0	0		0	0	0	1 (2)
Missing	0	0	0	0	0	1 (17)	0	0	0	1 (2)
ECOG PS at baseline										
0	0	1 (25)	3 (75)	2 (33)	1 (17)	4 (67)	4 (67)	2 (67)	2 (67)	19 (46)
1	3 (100)	3 (75)	1 (25)	4 (67)	5 (83)	2 (33)	2 (33)	1 (33)	1 (33)	22 (54)
Metastatic site nos., median (range)	3 (1-5)	3 (1-4)	2 (1-3)	2 (1-3)	2 (1-4)	2 (1-2)	3 (1-9)	2 (1-2)	1 (1-2)	2 (1-9)
Presence of visceral disease, *n* (%)	2 (67)	3 (75)	1 (25)	4 (67)	3 (50)	5 (83)	5 (83)	2 (67)	1 (33)	26 (63)

Supplementary Table S4. Adverse events during dose escalation (phase Ia) by preferred term for events with occurrence in >25% of total population

Preferred term, all grades<br>Grades 3-5, *n* (%)	100 mg QD (*n*= 3)	200 mg QD (*n*= 4)	400 mg QD (*n*= 4)	600 mg QD (*n*= 6)	600 mg QD (*n*=6) **Non-Fasting**	300 mg **BID** (*n*=6)	800 mg QD (*n*=6)	800 mg QD (*n*=3) **Non-Fasting**	400 mg **BID** (*n*=3)	All Patients (*N*=41)
**Diarrhea**	1 (33)	1 (25)	4 (100)	4 (67)	6 (100)	4 (67)	6 (100)	3 (100)	3 (100)	32 (78)
Grades 3-5	0	0	0	0	0	1 (17)	1 (17)	0	0	0
**Fatigue**	1 (33)	1 (25)	3 (75)	5 (83)	4 (67)	2 (33)	6 (100)	3 (100)	3 (100)	28 (68)
Grades 3-5	0	1 (25)	0	0	0	0	1 (17)	1 (33)	0	3 (7)
**Nausea**	1 (33)	3 (75)	2 (50)	6 (100)	2 (33)	4 (67)	6 (100)	2 (67)	2 (67)	28 (68)
Grades 3-5	0	1 (25)	0	0	0	0	0	0	0	1 (2)
**Anemia**	1 (33)	2 (50)	2 (50)	4 (67)	4 (67)	1 (17)	1 (17)	1 (33)	2 (67)	18 (44)
Grades 3-5	0	0	0	1 (17)	0	0	0	0	0	1 (2)
**AST increased**	2 (67)	1 (25)	0	4 (67)	3 (50)	4 (67)	1 (17)	0	2 (67)	17 (42)
Grades 3-5	0	0	0	1 (17)	0	1 (17)	0	0	0	2 (5)
**Constipation**	2 (67)	0	3 (75)	4 (67)	2 (33)	2 (33)	2 (33)	0	2 (67)	17 (42)
Grades 3-5	0	0	1 (25)	0	0	0	0	0	0	0
**Vomiting**	1 (33)	2 (50)	1 (25)	1 (17)	1 (17)	1 (17)	4 (67)	3 (100)	2 (67)	16 (39)
Grades 3-5	0	1 (25)	0	0	0	0	0	0	0	1 (2)
**ALT increased**	2 (67)	1 (25)	0	3 (50)	3 (50)	3 (50)	0	0	2 (67)	14 (34)
Grades 3-5	0	0	0	1 (17)	0	0	0	0	0	1 (2)
**Cough**	1 (33)	1 (25)	2 (50)	2 (33)	1 (17)	2 (33)	2 (33)	2 (67)	0	13 (32)
Grades 3-5	0	0	0	0	0	0	0	0	0	0
**Decreased appetite**	0	1 (25)	2 (50)	3 (50)	2 (33)	1 (17)	2 (33)	0	2 (67)	13 (32)
Grades 3-5	0	0	0	1 (17)	0	0	0	0	0	
**Abdominal pain**	2 (67)	1 (25)	3 (75)	2 (33)	1 (17)	1 (17)	1 (17)	0	1 (33)	12 (29)
Grades 3-5	0	1 (25)	0	0	0	0	0	0	0	1 (2)
**Arthralgia**	1 (33)	0	1 (25)	2 (33)	3 (50)	3 (50)	0	1 (33)	1 (33)	12 (29)
Grades 3-5	0	0	0	0	0	0	0	0	0	0

**Dyspepsia**	1 (33)	0	1 (25)	2 (33)	2 (33)	1 (17)	2 (33)	1 (33)	1 (33)	11 (27)
Grades 3-5	0	0	0	0	0	0	0	0	0	0
**Flatulence**	0	0	0	2 (33)	2 (33)	5 (83)	1 (17)	1 (33)	0	11 (27)
Grades 3-5	0	0	0	0	0	0	0	0	0	0
**Muscle spasms**	1 (33)	2 (50)	0	2 (33)	1 (17)	2 (33)	1 (17)	1 (33)	1 (33)	11 (27)
Grades 3-5	0	0	0	0	0	0	0	0	0	0

Supplementary Table S5. All serious adverse events regardless of attribution in the safety population in phase 1a (*n*=41)

Preferred term	*n* (%)	Grade	Cohorts
Pneumonia	2 (5%)	3	400 mg; 800 mg, non-fasting
Fracture	2 (5%)	3	400 mg; 300 mg, BID
Abdominal pain	1 (2%)	3	200 mg
Adrenal insufficiency	1 (2%)	3	800 mg, non-fasting
Anemia	1 (2%)	3	600 mg
Ascites	1 (2%)	3	600 mg
Blood bilirubin increased	1 (2%)	5	800 mg
Cardiac arrest	1 (2%)	4	600 mg, non-fasting
Cardiac failure	1 (2%)	5	600 mg, non-fasting
Fatigue	1 (2%)	3	800 mg, non-fasting
Hyponatremia	1 (2%)	4	600 mg, non-fasting
Metastases to peritoneum	1 (2%)	5	600 mg, non-fasting
Nausea	1 (2%)	3	200 mg
Pneumothorax	1 (2%)	1	800 mg, non-fasting
Presyncope	1 (2%)	2	300 mg, BID
Pulmonary embolism*<br>Skin infection	1 (2%)<br>1 (2%)	4<br>3	300 mg, BID<br>300 mg, BID
Thrombocytopenia	1 (2%)	4	600 mg
Urinary tract infection	1 (2%)	3	100 mg
Venous thrombosis	1 (2%)	3	300 mg, BID
Vomiting	1 (2%)	3	200 mg

*Related to study drug.

Supplementary Table S6. All serious adverse events regardless of attribution in the safety population in phase Ib and IIa (n=111)

Preferred term	n (%)	Grade	Cohorts
Pneumonia	2	3	A2, B1
	1	2	B1
Breast cancer	2	5	B1
Pulmonary embolism	2	3	B1, D1
Sepsis	2	4	B1, B2
Vomiting	2	3	A1 (both)
Abdominal pain	1	2	D1
Acute kidney injury	1	5	B1
Appendicitis perforated	1	2	D1
Ascites	1	3	A1
Atrial fibrillation	1	3	C1
Cardiac failure	1	3	B1
Deep vein thrombosis	1	2	B1
Diarrhea	1	3	B1
Dyspnea	1	3	B1
Endometrial hyperplasia	1	3	B1
Headache	1	3	B1
Hepatic hemorrhage	1	2	A1
Hip fracture	1	3	B1
Hydronephrosis	1	3	B1
Hypertension	1	3	A1
Hyponatremia	1	4	B1
Pain in extremity	1	2	D1
Pleural effusion	1	2	C1
Seizure	1	3	B1
Syncope	1	3	B1
Tumor pain	1	3	B1
Urinary tract infection	1	1	A1

Supplementary Figure

Supplementary Fig. S1 Study scheme and patient disposition for GDC-0810 single-agent in phase 1a dose escalation (*n*=41) and phase 2a dose expansion (*n*=101) studies. In phase 1b, GDC-0810 was combined with palbociclib (125 mg on days 1-21 of 28-day cycles) or luteinizing hormone releasing hormone (LHRH) agonist. AE = adverse event; CPD = clinical progressive disease; Non-CR/PD = non-complete response or non-progressive disease; PD = progressive disease; SD = stable disease; Unk = unknown

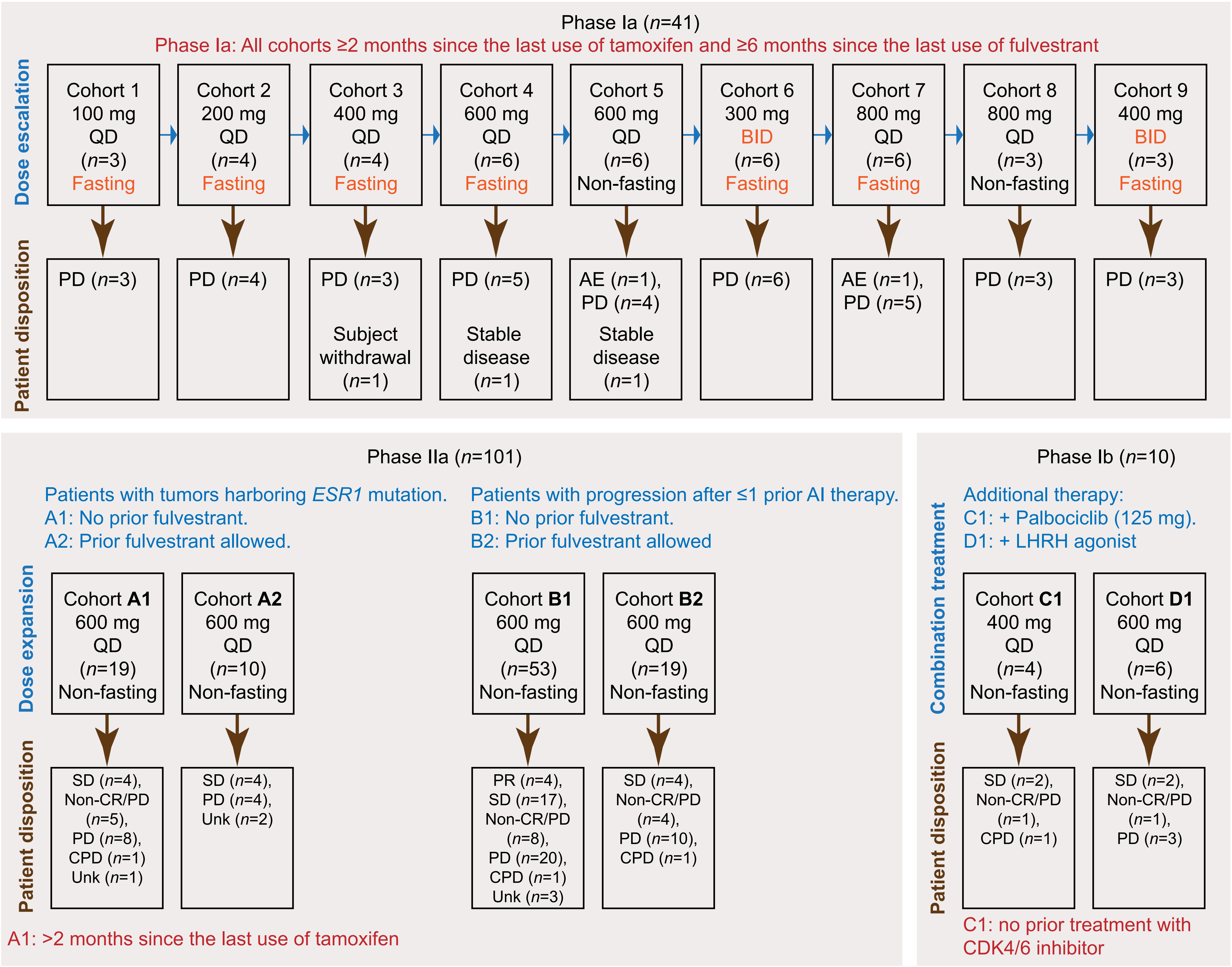

Phase Ia (n=41)
Phase Ia: All cohorts ≥2 months since the last use of tamoxifen and ≥6 months since the last use of fulvestrant
Dose escalation
Patient disposition
Cohort 1 100 mg QD (n=3) Fasting
Cohort 2 200 mg QD (n=4) Fasting
Cohort 3 400 mg QD (n=4) Fasting
Cohort 4 600 mg QD (n=6) Fasting
Cohort 5 600 mg QD (n=6) Non-fasting
Cohort 6 300 mg BID (n=6) Fasting
Cohort 7 800 mg QD (n=6) Fasting
Cohort 8 800 mg QD (n=3) Non-fasting
Cohort 9 400 mg BID (n=3) Fasting
PD (n=3)
PD (n=4)
PD (n=3) Subject withdrawal (n=1)
PD (n=5) Stable disease (n=1)
AE (n=1), PD (n=4) Stable disease (n=1)
PD (n=6)
AE (n=1), PD (n=5)
PD (n=3)
PD (n=3)
Phase IIa (n=101)
Patients with tumors harboring ESR1 mutation.
A1: No prior fulvestrant.
A2: Prior fulvestrant allowed.
Patients with progression after ≤1 prior AI therapy.
B1: No prior fulvestrant.
B2: Prior fulvestrant allowed
Dose expansion
Patient disposition
Cohort A1 600 mg QD (n=19) Non-fasting
Cohort A2 600 mg QD (n=10) Non-fasting
Cohort B1 600 mg QD (n=53) Non-fasting
Cohort B2 600 mg QD (n=19) Non-fasting
SD (n=4), Non-CR/PD (n=5), PD (n=8), CPD (n=1) Unk (n=1)
SD (n=4), PD (n=4), Unk (n=2)
PR (n=4), SD (n=17), Non-CR/PD (n=8), PD (n=20), CPD (n=1) Unk (n=3)
SD (n=4), Non-CR/PD (n=4), PD (n=10), CPD (n=1)
A1: >2 months since the last use of tamoxifen
Phase Ib (n=10)
Additional therapy:
C1: + Palbociclib (125 mg).
D1: + LHRH agonist
Combination treatment
Patient disposition
Cohort C1 400 mg QD (n=4) Non-fasting
Cohort D1 600 mg QD (n=6) Non-fasting
SD (n=2), Non-CR/PD (n=1), CPD (n=1)
SD (n=2), Non-CR/PD (n=1), PD (n=3)
C1: no prior treatment with CDK4/6 inhibitor
